# Laparoscopic resection of a huge mature cystic teratoma of the right adrenal gland through retroperitoneal approach: a case report and literature review

**DOI:** 10.1186/s12957-015-0734-z

**Published:** 2015-11-18

**Authors:** Hengping Li, Tao Zhao, Qiang Wei, Haichao Yuan, Dehong Cao, Pengfei Shen, Liangren Liu, Hao Zeng, Ni Chen

**Affiliations:** Department of Urology, Gansu Provincial Hospital, Lanzhou, Gansu People’s Republic of China; Department of Urology, West China Hospital, Sichuan University, Chengdu, Sichuan People’s Republic of China; Department of Urology, Shenzhen Hospital, Peking University, Shenzhen, Guangdong People’s Republic of China; Department of Pathology, West China Hospital, Sichuan University, Chendu, Sichuan People’s Republic of China

**Keywords:** Mature teratoma, Adrenal tumour, Laparoscopy

## Abstract

**Background:**

At present, the primary mature cystic teratoma in the adrenal gland is extremely rare in adults, according to the literature. In addition, a completely retroperitoneoscopic resection of mature cystic teratomas has been reported only in two cases.

**Case presentation:**

We report a case of a large mature cystic teratoma with a regular margin in the right adrenal gland. Three months before surgery, abdominal enhanced computer tomography revealed a 5.7 × 4.9 × 4.3 cm lipoid tumour of mixed density with calcification in the tumorous centre, clinically diagnosed as adrenal myelolipoma or adenoma. Retroperitoneoscopic adrenalectomy was successfully performed; however, the tumour had increased in size to approximately 6.0 × 7.0 × 11 cm. The pathological report suggested the final diagnosis of mature cystic teratoma. The patient had an uneventful course after the surgery and was free of recurrence or metastasis within 8 months of follow-up.

**Conclusions:**

Retroperitoneoscopic adrenalectomy for large adrenal masses is safe and feasible. To the best of our knowledge, this is the first report where of a large mature cystic teratoma of the right adrenal gland has been completely resected using retroperitoneoscopic approach.

## Background

Teratomas are germ cell tumours mainly originating from the testes and ovaries containing more than one or usually all three germ layers. Teratomas are categorised as mature with malignant potential and immature or malignant. The extragonadal locations account for 15 % of all teratomas, the retroperitoneum is the least common site [1] and the primary mature cystic teratoma in the adrenal gland is extremely rare in adults, according to the literature [2, 3]. In addition, a completely laparoscopic resection of mature cystic teratomas through transperitoneal approach has been reported only in two cases [4]. Here, we present an extremely rare case of a huge mature cystic teratoma in the right adrenal gland resected using completely laparoscopic adrenalectomy through retroperitoneal approach in an adult.

## Case presentation

**Fig. 1:**
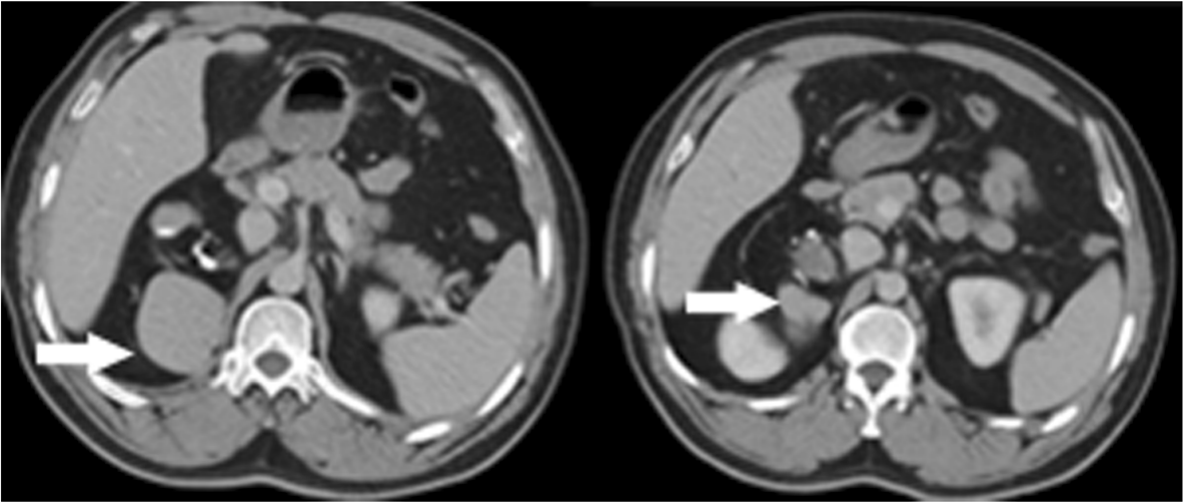
Abdominal enhanced computer tomography demonstrated a 5.7 × 4.9 × 4.3 cm lipoid tumour of mixed density and nodes of high density with calcification in the tumorous centre (*arrows* showing tumour of mixed density)

**Fig. 2:**
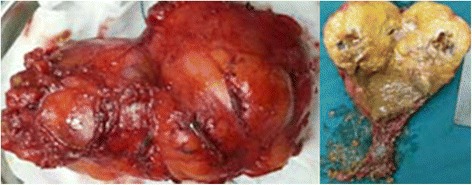
Gross photograph of the huge tumour measuring approximately 6.0 × 7.0 × 11 cm, photography of cut-face manifesting a cystoma with wall thickness of 0.1–0.5 cm accompanied with brown fluid

**Fig. 3:**
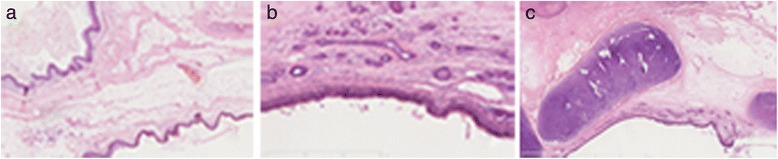
On microscopic examination, the tumour was mainly composed of the mature fat tissue but also contained a cystoma lined with squamous cell developed from skin tissue (**a**) and ciliated columnar epithelial cell arising from respiratory tract (**b**); the cartilage and hair were present in the cystic centre (**c**), haematoxylin and eosin, ×200

An asymptomatic 49-year-old male was referred to our hospital because an abdominal mass in place of right adrenal region was incidentally discovered in physical checkup. Ultrasonography showed a hypoechoic tumour with a regular margin measuring 5.2 × 4.6 × 4.0 cm, and abdominal enhanced computer tomography (CT) revealed a 5.7 × 4.9 × 4.3 cm lipoid tumour of mixed density with calcification in the tumorous centre (Fig. 1). Blood and urine test results for hormones of adrenal gland, alpha-fetoprotein (AFP) and human chorionic gonadotropin (hCG) were unremarkable. There were no history of other diseases except for chronic hepatitis B; therefore, the clinical diagnosis was adrenal myelolipoma or adenoma. Subsequently, the completely retroperitoneoscopic adrenalectomy was successfully performed, but the surgical process was quite difficult due to the size of the tumour and relatively confined space of the retroperitoneum. The process of the surgery as follows: The patient was placed in a standard left lateral decubitus position, and the table was flexed. A 2-cm transverse incision was made posteriorly, below the 12th rib and lateral to the erector spinae muscules and deepened down to the lumbodorsal fascia. This fascia was incised, the finger is inserted into the retroperitoneal space to dissect retroperitoneal fascia and expand the retroperitoneal space, two 10-mm ports inserted above the iliac crest and below the tip of the 12th rib, respectively, and a 5-mm ports inserted posterior axillary line, below the 12th rib and fixed with a silk suture for the prevention of gas leakage. On inserting of the laparoscope into the port above the iliac crest, extraperitoneal fat were excised and the Gerota’s fascia was incised, the tumour was appeared and proceed with the dissection along with the surface of the tumour, till tumour completely exposed and resected, at last, the tumour was placed in the endo bag and puncture cystoma for removal of tumour measured approximately 6.0 × 7.0 × 11 cm after resection. It had increased in size after initial discovery because the patient had not consent for the surgery before 3 months. Malignancy was suspected in the process of the surgery on the basis of the increase of shape over short period, but the tumour lightly adhered to surrounding tissue and organ; hence, only the right adrenal gland was invaded by the tumour. Duration of the surgery was 125 min; no bleeding occurred during the surgery, and the patient was discharged after 72 h. On gross examination after the surgery, the thin capsule of tumour specimen with dust colour was found to be intact; the cut-face manifested a cystoma with wall thickness of 0.1–0.5 cm accompanied with cartilage, hair, calcification and a solid yellow lipid, and the residual adrenal tissue measured 1 cm in diameter (Fig. 2). On microscopic examination, the tumour was found to be mainly composed of mature fat tissue, but it also contained a cystoma lined with squamous cells developed from skin tissue and ciliated columnar epithelial cells arising from the respiratory tract; cartilage and hair were present in the cystic centre (Fig. 3). Some normal adrenal gland tissue was also observed in the tumorous margin. According to these findings, the pathological analysis of the specimen from the right adrenal gland suggested cystic teratoma. The patient had an uneventful course after surgery and was free of recurrence after 8 months of follow-up.

### Discussion

The mature cystic teratoma is a neoplasm developed from totipotential cells. Although it mainly occurs in the gonads, the cases of extragonadal teratomas, such as anterior mediastinum, sacrococcygeal region and retroperitoneum, have been reported in the literature [5]. The primary mature cystic teratoma in the adrenal gland is extremely rare. The few existing reports reveal that retroperitoneal teratoma is more common in females and children [6] and is generally detected on the left side [7]. We reported a case of a primary mature cystic teratoma in the right adrenal gland in an adult with no any signs or symptoms and with unremarkable outcome of laboratory testing for adrenal hormone, AFP and hCG. Primary tumours metastasizing to this region have been reported in the breasts and gonads. To rule out the possible metastasis in the right adrenal gland, all the original radiological records were carefully reviewed, and no evidence of metastasis in the testes or other parts in the body was found.

The diagnosis of adrenal teratoma depends on radiological examination and pathological analysis. Adrenal teratomas present with vague symptoms, such as abdominal distention, dull flank pain, epigastric pain and even intestinal obstruction caused by compression of the neoplasm [8]. There were no remarkable symptoms observed in our case despite the presence of a large mass. The patient could have been asymptomatic because it was a columnar mass along the longitudinal axis of the kidney; therefore, increasing mass did not cause distention of the renal capsule. On the abdominal CT scan, mostly fatty components with less calcification were observed in the region of right adrenal gland with the absence of normal adrenal gland tissue, clinically diagnosed as adrenal myelolipoma or adenoma. The presence of calcification in a teratoma would not necessarily imply benignity because 12.5 % of the calcified teratomas are malignant [9]. The majority of mature teratomas in the retroperitoneum are benign neoplasms, and only 26 % are malignant [10]. The pathological criteria for benign lesion of mature cystic teratoma are (1) absence of malignant or immature elements in the tumour, (2) absence of other similar lesions in other parts of the body, (3) normal serum levels of AFP and hCG and (4) absence of recurrence on long-term follow-up [4]. The diagnostic criteria in the present case are completely in accordance with previously described cases.

To date, surgery is the method of choice in the treatment of the mature teratoma. Open surgery was usually performed for mature cystic teratomas according to the reviewed reports, with the exception of completely laparoscopic surgery in two cases through the transperitoneal approach [4] and of hand-assisted laparoscopy converted in the mid of laparoscopic surgery in another case [11]. In our case, the completely laparoscopic surgery through retroperitoneal approach was successful at first attempt, although the operative process was difficult due to the relatively confined space of the retroperitoneum and the large size of the mass. To the best of our knowledge, this is the first report where of a large mature cystic teratoma of the right adrenal gland has been completely resected using laparoscopy through retroperitoneal approach.

The mature cystic teratoma is a neoplasm with malignant potential. In the cases of retroperitoneal teratomas, the malignancy rate of 25.8 % in adults is significantly higher than the 6.8 % rate documented in children [3]. Despite the benign nature of mature cystic teratoma, close follow-up is recommended because the incidence of malignant transformation is approximately 3–6 % [11]. The patient in the present report was free of recurrence and metastasis within 8 months post-surgery and is doing well.

## Conclusions

Mature cystic teratoma of the adrenal gland amongst adults is quite rare, particularly on the right side. Retroperitoneoscopic adrenalectomy for large adrenal masses is safe and feasible. To the best of our knowledge, this is the first report where only laparoscopy through retroperitoneal approach was used for treating a huge mature cystic teratoma.

### Consent

Written informed consent was obtained from the patient for publication of this case report and any accompanying image.

## Abbreviations

AFP: alpha-fetoprotein
CT: computer tomography
hCG: human chorionic gonadotropin

## References

[1] Bedri S, Erfanian K, Schwaitzberg S, Tischler AS (2002). Mature cystic teratoma involving adrenal gland. Endocr Pathol.

[2] Otani M, Tsujimoto S, Miura M (2001). Intrarenal mature cystic teratoma associated with renal dysplasia: case report and literature review. Pathol Int.

[3] Gatcombe HG, Assikis V, Kooby D (2004). Primary retroperitoneal teratomas: a review of the literature. J. Surg. Oncol.

[4] Octavio A, Castillo GV, Villeta M, Leonardo A, Oscar S (2006). Laparoscopic resection of adrenal teratoma. JSLS.

[5] Hui JPK, Luk WH, Siu CW, Chan JCS (2004). Teratoma in the region of an adrenal gland in a 77-year-old man. JHK Coll Radio.

[6] Li Y, Zhong Z, Zhao X (2011). Primary mature teratoma presenting as an adrenal tumor in a child. Uro.

[7] Luo CC, Huang CS, Chu SM, Chao HC, Yang CP, Hsueh C (2005). Retroperitoneal teratomas in infancy and childhood. Pediatr Surg Int.

[8] Ratan SK, Ratan J, Kalra R (2002). Large benign cystic teratoma of the mesosigmoid causing intestinal obstruction: report of a case. Surg Today.

[9] Bruneton JN, Diard F, Drouillard JP, Sabatier JC, Tavernier JF (1980). Primary retroperitoneal teratoma in adults: presentation of two cases and review of the literature. Radiology.

[10] Scott AL, Abbassi-Ghadi N, Archer CM, Swamy R, Gupta S (2010). Neuroendocrine carcinoma arising within a retroperitoneal mature teratoma. Ann R Coll Surg Engl.

[11] Fuminori S, Hiromitsu M, Kenichi M (2010). Primary retroperitoneal mature cystic teratoma presenting as an adrenal tumor in an adult. Int J Uro.

